# Allelopathy and Allelochemicals of *Solidago canadensis* L. and *S. altissima* L. for Their Naturalization

**DOI:** 10.3390/plants11233235

**Published:** 2022-11-25

**Authors:** Hisashi Kato-Noguchi, Midori Kato

**Affiliations:** Department of Applied Biological Science, Faculty of Agriculture, Kagawa University, Miki, Kagawa 761-0795, Japan

**Keywords:** allelochemical, invasive species, monospecific stand, mycorrhizal colonization, *Solidago*, phytotoxicity, rhizosphere soil

## Abstract

*Solidago canadensis* L. and *Solidago altissima* L. are native to North America and have naturalized many other continents including Europa and Asia. Their species is an aggressive colonizer and forms thick monospecific stands. The evidence of the allelopathy for *S. canadensis* and *S. altissima* has accumulated in the literature since the late 20th century. The root exudates, extracts, essential oil and rhizosphere soil of *S. canadensis* suppressed the germination, growth and the arbuscular mycorrhizal colonization of several plants, including native plant species. Allelochemicals such as fatty acids, terpenes, flavonoids, polyphenols and their related compounds were identified in the extracts and essential oil of *S. canadensis*. The concentrations of total phenolics, total flavonoids and total saponins in the rhizosphere soil of *S. canadensis* obtained from the invasive ranges were greater than those from the native ranges. Allelochemicals such as terpenes, flavonoids, polyacetylene and phenols were also identified in the extracts, essential oil and the rhizosphere soil in *S. altissima.* Among the identified allelochemicals of *S. altissima,* the *cis*-dehydromatricaria ester may be involved in the allelopathy considering its growth inhibitory activity and its concentration in the rhizosphere soil. Therefore, the allelopathy of *S. canadensis* and *S. altissima* may support their invasiveness, naturalization and formation of thick monospecific stands. This is the first review article focusing on the allelopathy of both of *S. canadensis* and *S. altissima*.

## 1. Introduction

*Solidago canadensis* sensu lato (s.l.), belonging Asteraceae, is an erect rhizomatous perennial plant, native to North America. *S. canadensis* s.l. was introduced to Europe as an ornamental plant in the 17th to 18th centuries. The species spread from the gardens to the natural environments, and has extended its habitats in Central and Eastern Europe. It expanded its habitat at a rate of 741 km^2^ per year in Europe [1]. The species has also been introduced and naturalized in many other countries such as Australia, Brazil, China, India, New Zealand and Japan [2,3,4,5,6].

**Figure 1:**
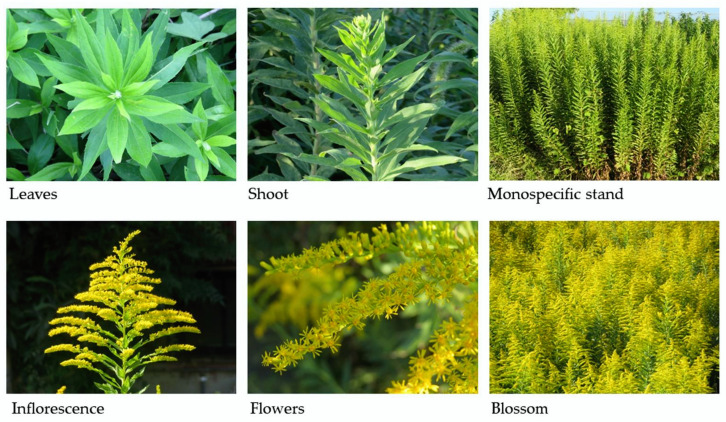
*Solidago canadensis* s.l.

The species expands its habitat though seed distribution and rhizome expansion. The rhizomes arise near the base of the shoots in autumn and produce aerial stems from their apex in the following spring. The stems are not branched, and bear triple-nerved, lanceolate, alternate leaves which are found along the stems and roots at the base of the shoots. The rhizome systems contribute to expanding the species’ community and to form thick monospecific stands [7,8]. Shoot density in the established stands of the species was reported to be 309 shoots per m^2^ [3]. In addition, oil-filled cavities, which contain terpenes and/or lipids, were randomly distributed in the rhizomes [9]. These compounds may have some biological functions such as allelopathy. The species is a prolific seed producer. Its inflorescence forms broad pyramidal panicles, which contain numerous florets (Figure 1). A single plant produces 1000–20,000 light-winged achenes which contain seeds. The achenes disperse easily by wind, water and human activities. The germination rate is 30–75%, depending on the conditions [7,8,10,11]. The seed distribution may contribute to establishing the populations of *S. canadensis* s.l. in new habitats.

This species has adapted to a wide range of soil fertility and water potential [8,11,12], as well as been colonized into disturbed areas such as abandoned fields, roadsides, riverbanks and forest edges [11,13,14]. The species established its population on an agriculture field in two years after the abandonment [15,16]. Once established, the population remained dominant over 30 years [8,17,18,19,20]. It was also reported that the species showed great impact on the native plant diversity in the introduced ranges [21,22,23]. Owing to the potential of the species for the rapid expansion and the formation of thick monospecific stands in the introduced ranges, as well as its impact on the environments, *S. canadensis* s.l. has been designated as a harmful invasive plant species [4,24].

The *S. canadensis* complex is a highly variable species. *S. canadensis* s.l. contains *S. canadensis* L. as *S. canadensis* subsp. *canadensis* (L.), and *S. altissima* L. as *S. canadensis* subsp. *altissima* (L.) O.Bolòs et Vigo [2,25,26]. *S. canadensis* and *S. altissima* are very similar taxa. The field experiments also showed that the competitive abilities of *S. altissima* and *S. canadensis* against other plant species was similar [27]. However, they could be distinguished by their morphological traits such as shoot length and flowering time [2,26,28,29]. The chromosome number between *S. canadensis* (diploid; 2*n* = 18) and *S. altissima* (hexaploid; 2*n* = 54) is also different [7,29,30]. The native ranges of both species in North America are not exactly the same [8,26]. In addition, the experimental crossing of *S. canadensis* and *S. altissima* could not bear viable seeds [30], which indicates a genetic barrier between both taxa. However, owing to the lack of consistency in the identification of the species, the separation of both species has been considered to be very problematic [2,28,29]. For example, most European populations of both species were described to be *S. altissima* [3,25], although macro-morphological analyses indicate that *S. canadensis* is a common species in Europa [29,31]. *S. altissima* was also mentioned as a synonym of *S. canadensis* [32,33]. There may have been a misidentification of the species.

It was reported that invasive plants are often allelopathic and inhibit the germination and growth of the native plant species in the invasive ranges through specific secondary metabolites defined as allelochemicals [34,35,36,37,38,39,40,41,42,43]. *S. canadensis* showed an allelopathic activity on the sugar maple seedlings in the field, greenhouse and laboratory conditions [44]. The *cis*-Dehydromatricaria ester was identified in the extracts of *S. altissima* as its allelochemical [45]. The evidence of the allelopathy of *S. canadensis* and *S. altissima* has been accumulated since the late 20th century, and their allelopathy was often implicated in the potential of their invasiveness and naturalization. However, there has been no review paper focusing on the allelopathy of both *S. canadensis* and *S. altissima*. This review provides an overview of the allelopathy and allelochemicals in *S. canadensis* and *S. altissima*, as well as a discussion on the involvement of allelopathy in the invasiveness and naturalization of the species. Despite the potential misidentification of *S. canadensis* and *S. altissima* described earlier, due to the fact that both species are very similar and the identification of the species is problematic [2,28,29], this paper followed the identification of the species in the publications because it is impossible to confirm their identification.

## 2. Allelopathy of *S. canadensis*

Allelopathy is the chemical interaction between donor plants and recipient plants through allelochemicals. Allelochemicals are produced in some plant parts and released into the vicinity of the donor plants, including their rhizosphere soil either by the root exudation, rainfall leachates, volatilization from the plant parts or decomposition processes of plant residues [46,47,48,49]. Several investigations in field conditions showed that *S. canadensis* reduced the number and biodiversity of the native plant community in its invaded ranges [50]. The invasion level of *S. canadensis* correlated negatively with the taxonomic diversity of the native plant community, and positively with the invasibility of the community [51]. Those observations may imply the involvement of allelopathy in the interaction between *S. canadensis* and native plant species to some extent. Many researchers have evaluated the allelopathic activity of the root exudates, rhizosphere soil, residues and plant extracts of *S. canadensis* (Table 1).

### 2.1. Allelopathy of Root Exudate and Plant Residue

Root exudates of *S. canadensis*, which were obtained from its aeroponic culture, significantly suppressed the growth of two Asian original plant species; *Gnaphalium affine* D.Don and *Xanthium sibiricum* Patrin ex Widder., two of America origin; *Conyza canadensis* (L.) Cronquist and *Celosia argentea* L., two of tropical origin; *Aster subulatus* Michx. and *Sesbania cannabina* (Renz.) Poir. and a cosmopolitan species; *Eclipta prostrata* (L.) L. The suppression rate was similar in all plant species [52]. Root exudates of *S. canadensis* also showed the growth inhibition of *Arabidopsis thaliana* (L.) Heynh. [53]. When the seeds of seven European native plant species were sown into the *S. canadensis* cultivated soils with or without activated carbon, the germination of five species such as *Dactylis glomerata* L., *Lythrum salicaria* L., *Stachys officinalis* (L.) Trevis. and *Trifolium pratense* L. were significantly suppressed in activated carbon-free plots than in activated carbon plots. Although the germination rate was not significantly different between both plots, the biomass of *Arrhenatherum elatius* (L.) P.Beauv. ex J. et C.Presl in the activated carbon plots after three months of sowing was two times greater than that in the activated carbon-free plots [53]. Activated carbon is a widely used material to investigate allelopathy because it adsorbs allelochemicals in the plant rhizosphere soil [34,54]. In addition, aqueous extracts of the rhizosphere soil of *S. canadensis* inhibited the germination and growth of *Digitaria sanguinalis* (L.) Scop. and *Amaranthus retroflexus* L., and the inhibitory activity was greater in the extracts of the soil obtained from the invasive ranges of *S. canadensis* (China) than that from its native ranges (USA) [55]. These observations suggest that certain allelochemicals, which may cause growth inhibition, would be released into the rhizosphere soil as root exudates of *S. canadensis*, and the released allelochemicals in the soil may be greater in the invasive ranges than those in the native ranges.

Crushed stems, leaves and rhizomes of *S. canadensis* were mixed with soil and water and kept at 20/15 °C (12/12 h light/dark condition), and the mixture was filtered after 45 days. The obtained filtrate suppressed the germination and growth of *Raphanus sativus* L. and *Triticum aestivum* L. [56]. This observation also suggests that certain allelochemicals may be released into the rhizosphere soil during the decomposition process of plant residues of *S. canadensis*.

### 2.2. Allelopathy of Plant Extract

Some plant tissues may contain allelochemicals, since allelochemicals are synthesized and stored in certain plant tissues until their release into the environment [46,47,48,49]. Many investigations on the allelopathic activity of the extracts from different plant parts of *S. canadensis* have been conducted. Aqueous extracts of the leaves of *S. canadensis* inhibited the germination and root growth of *Raphanus sativus* L. and *Lactuca sativa* L. [57], as well as those of *Triticum aestivum* L. and *Setaria viridis* (L.)P.Beauv. [58]. The extracts also suppressed the germination, growth and chlorophyll content of *Trifolium pratense* L. and *Raphanus sativus* L., and increased their electrolyte leakage from the cell membrane of the seedlings [59,60].

The fresh leaves and stems of *S. canadensis* were soaked in water for 48 h, and the obtained soaking water showed the inhibitory activity on the germination and growth of *Raphanus sativus* L. and *Triticum aestivum* L. [54]. Aqueous extracts of the above-ground parts of *S. canadensis* suppressed the germination and growth of *Lactuca sativa* L. [61], as well as those of *Digitaria sanguinalis* (L.) Scop. and *Amaranthus retroflexus* L. [55]. The inhibitory activity was greater in the plant extracts obtained from the heavily invaded stands than in those obtained from the lightly invaded stands [62], and in the plant extracts obtained from the invasive ranges than those from the native ranges [55].

Aqueous extracts of the above-ground parts and roots of *S. canadensis* inhibited the germination and growth of *Zoysia japonica* Steud, and the extracts of the above-ground parts significantly stimulated malondialdehyde and peroxidase activity [62]. The extracts of the stems, roots, blossoms and seeds of *S. canadensis* suppressed the germination and growth of *Brassica napus* L. and *Lolium perenne* L. [63], and the extracts of the roots and rhizomes of *S. canadensis* also inhibited the root growth of *Raphanus sativus* L. and *Lactuca sativa* L. [57].

Aqueous ethanol extracts of the roots and rhizomes of *S. canadensis* inhibited the germination and growth of *Trifolium repens* L., *Trifolium pratense* L., *Medicago lupulina* L., *Suaeda glauca* (Brunge) Brunge, *Plantago virginica* L., *Kummerowia stipulacea* (Maxium.) Makino, *Festuca arundinacea* Schreb., *Ageratum conyzoides* L., *Portulaca oleracea* L. and *Amaranthus spinosus* L. [64]. Aqueous ethanol extracts of the above- and below-ground parts of *S. canadensis* suppressed the germination of *Kummerowia striata* (Thunb.) Schindl., and the inhibitory activity was greater in the plant extracts collected from the invasive ranges of *S. canadensis* than those from its native ranges [65]. Aqueous and ethanol extracts of the leaves, stems and rhizomes of *S. canadensis* inhibited the germination and growth of *Morus alba* L., *Pharbitis nil* (L.) Roth, *Triticum aestivum* L. and *Brassica campestris* L., and the inhibition was grater in the ethanol extracts than in the aqueous extracts [66].

Investigations on the aqueous and ethanol extracts of every part of *S. canadensis* showed the allelopathic activity on the germination, growth, chlorophyll content, electrolyte leakage and/or some enzyme activities of several plant species, including the native plant species. The inhibitory activity was greater in the plant extracts obtained from the invasive ranges of *S. canadensis* than in those from its native ranges, and in the extracts collected from the heavily invaded stands than in those collected from the lightly invaded stands. These observations suggest that whole parts of *S. canadensis* may contain water and ethanol extractable allelochemicals, which may cause the inhibition. In addition, the plants grown in the invasive ranges and heavily invaded stands may contain more allelochemicals than the plants in the native ranges and lightly invaded stands.

### 2.3. Effects of the Extract on Arbuscular Mycorrhizal Fungi

The rhizomes of *S. canadensis* were soaked in water for 24 h, and the obtained soaking water caused the suppression of the arbuscular mycorrhizal colonization of *Echinochloa crus-galli* (L.) P.Beauv., *Kummerowia striata* (Thnb.) Schindl. and *Ageratum conyzoides* L. [67]. The field and greenhouse investigations also showed that *S. canadensis* altered the composition of the arbuscular mycorrhizal fungal population in its rhizosphere soil through the inhibition of some dominant species and the stimulation of other species. The established arbuscular mycorrhizal community increased the competitive ability and the biomass of *S. canadensis* [67,68,69,70,71]. This altered arbuscular mycorrhizal community also increased the mycorrhizal-mediated ^15^N uptake in *S. canadensis*, as well as decreased the ^15^N uptake in the native species *Kummerowia statrica* (Thunb.) Schindl. [72]. In addition, the aqueous ethanol extract of the roots and rhizomes of *S. canadensis* also suppressed the population of the soilborne pathogens, namely *Pythium ultimum* Trow and *Rhizoctonia solani* J.G. Kühn [73]. These observations indicate that the aqueous extracts *of S. canadensis* may alter the arbuscular mycorrhizal population and suppress the colonization of the native plant species. The established arbuscular mycorrhizal community enhanced the competitive ability of *S. canadensis*. Certain compounds in the extracts may be involved in the alteration of the arbuscular mycorrhizal community.

**Table 1:** Allelopathic activities of exudates, rhizosphere soil, residues and plant extracts of *S. canadensis*.

Source	Inhibition				Target Plant Species	Reference
	Germination	Growth	Chlorophyll	Mycorrhizal colonization		
**Root exudate**		✓			*Gnaphalium affine*, *Xanthium sibiricum*, *Conyza canadensis*, *Celosia argentea*, *Aster subulatus*, *Sesbania cannabina*, *Eclipta prostrata*	[52]
		✓			*Arabidopsis thaliana*,	[53]
**Rhizosphere soil**	✓	✓			*Dactylis glomerata*, *Lythrum salicaria*, *Stachys officinalis*, *Trifolium pratense*	[53]
**Soil extract**	✓	✓			*Digitaria sanguinalis*, *Amaranthus retroflexus*	[55]
**Residue**	✓	✓			*Raphanus sativus*, *Triticum aestivum*	[56]
**Plant extract**						
Whole part	✓	✓			*Kummerowia striata*	[65]
Leaf	✓	✓			*Raphanus sativus*, *Lactuca sativa*	[57]
	✓	✓			*Triticum aestivum*, *Setaria viridi*	[58]
			✓		*Raphanus sativus*	[59]
			✓		*Trifolium pratense*	[60]
Leaf and stem	✓	✓			*Raphanus sativus*, *Triticum aestivum*	[56]
Above-ground part	✓	✓			*Lactuca sativa*	[61]
	✓	✓			*Digitaria sanguinalis (L.) Scop. and Amaranthus retroflexus*	[55]
Above-ground part, root	✓	✓			*Zoysia japonica*	[62]
Stem, root, blossom, seed	✓	✓			*Brassica napus*, *Lolium perenne*	[63]
Leaf, stem, rhizome	✓	✓			*Morus alba*, *Pharbitis nil*, *Triticum aestivum*, *Brassica campestris*	[66]
Root, rhizome		✓			*Raphanus sativus*, *Lactuca sativa*	[57]
	✓	✓			*Trifolium repens*, *Trifolium pratense*, *Medicago lupulina*, *Suaeda glauca*, *Plantago virginica*, *Kummerowia stipulacea*, *Festuca arundinacea*, *Ageratum conyzoides*, *Portulaca oleracea*, *Amaranthus spinosus*	[69]
Rhizome				✓	*Echinochloa crus-galli*, *Kummerowia striata*, *Ageratum conyzoides*	[67]

### 2.4. Allelochemicals

As described above, the inhibitory activity of the extracts of the plants and rhizosphere soil of *S. canadensis* obtained from the invasive ranges was greater than that obtained from the native ranges [55,65]. The concentrations of total phenolics, total flavones and total saponins in *S. canadensis* and its rhizosphere soil obtained from the invasive ranges were also greater than those from the native ranges [55,65]. These concentrations in the soil obtained from *S. canadensis*-infested stands were also greater than those in the soil obtained from *S. canadensis*-free stands [74].

**Figure 2:**
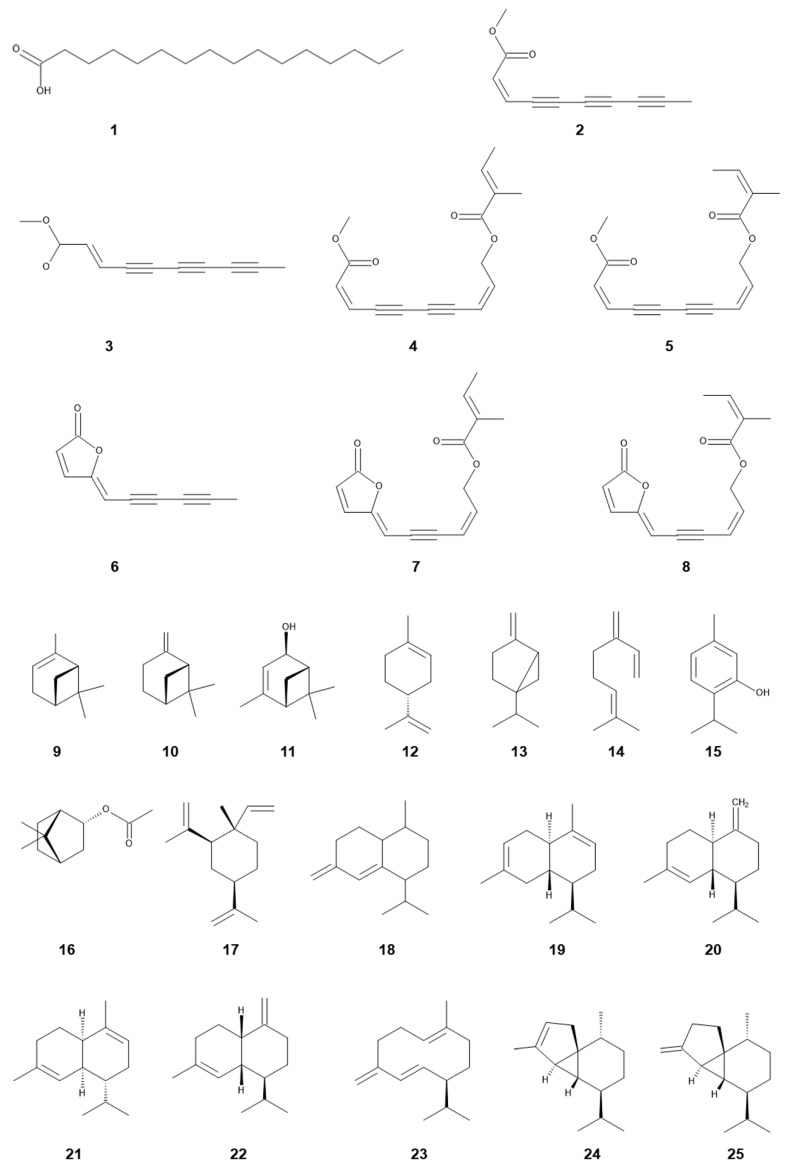

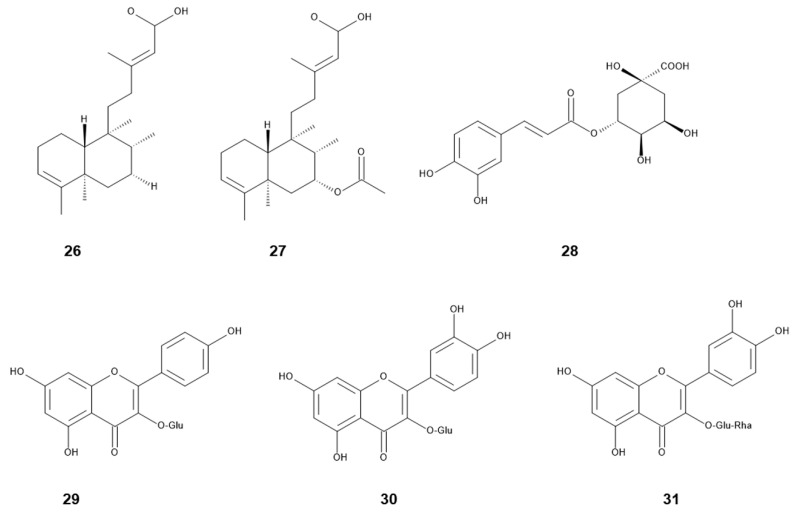
Allelochemicals identified in *S. canadensis* and *S. altissima*. Compound number and name were listed in Table 2.

Major compounds identified in the aqueous methanol extracts of the leaves and inflorescences of *S. canadensis* were chlorogenic acid, quercitrin and rutin (quercetin-3-*O*-β-rutinoside) (Figure 2) [75]. A fatty acid, *n*-hexadecanonic acid, was isolated from the aqueous ethanol extract of the stems and leaves of *S. canadensis* as an allelopathic agent. *n*-Hexadecanonic acid significantly inhibited the growth of *Triticum aestivum* L. [56]. A flavonoid, kaempferol-3-*O*-d-glucoside, was isolated from the aqueous ethanol extract of the *S. canadensis* straw, and the compound inhibited the growth of *Echinochloa colona* (L.) Link [75]. In addition, the concentration of rutin in the leaves of *S. canadensis* was greater than that of other *Solidago* species [76,77]. Some flavonoids were also identified in the aerial parts of *S. canadensis* [78].

The essential oil of many plant species was reported to have several biological activities, and to comprise volatile compounds such as terpenoids [79]. These volatile compounds often exhibited several biological activities including allelopathic activity [80,81]. Therefore, the involvement of the essential oil of *S. canadensis* in allelopathy was also investigated. The production of the essential oil was greater in *S. canadensis* obtained from the heavily invaded stands than that from mildly invaded stands [82]. The main components of the essential oil in the leaves and inflorescences of *S. canadensis* were α-pinene, trans-verbenol, limonene, bornyl acetate and β-cubebene [83], and those in the aerial parts of *S. canadensis* were α-pinene, β-pinene, germacrene D, limonene, thymol, (+)epi-bicyclosesquiphellandrene, β-cadinene, γ-cadinene, δ-cadinene, α-muurolene, γ-muurolene, α-cubebene and β-elemene [84,85]. The essential oil in the inflorescences of *S. canadensis* showed antimicrobial activity, antioxidant activity and free-radical scavenging activity, and its main components were α-pinene, germacrene D and limonene [86]. α-Pinene and β-pinene were reported to disturb the cell division through the interference of DNA synthesis, and cause membrane peroxidation [87,88,89]. Several monoterpenes were reported to alter soil microflora [90]. It was also reported that the essential oil of the aerial parts of *S. canadensis* inhibited the germination and radical growth of *Raphanus sativus* L. and *Lepidium sativum* L. [84].

### 2.5. Contribution of Allelopathy of S. canadensis to Its Invasiveness

All parts of the extracts of *S. canadensis,* including its rhizosphere soils, the root exudates, residues and essential oil, inhibited the germination and growth of several plant species (Table 1). These observations suggest that *S. canadensis* produces and accumulates allelochemicals in its plant tissues, and that it releases those allelochemicals into the surrounding environments of *S. canadensis,* including its rhizosphere soils through the root exudation, decomposition process of plant residues and volatilization from its essential oil. Several allelochemicals such as fatty acids, terpenes, flavonoids and polyphenols (Table 2) were identified in the extracts and essential oil of *S. canadensis.* Some of these compounds were reported to inhibit the germination and growth of other plants species, including the native plant species.

The extracts of *S. canadensis* and its rhizosphere soil obtained from its invasive ranges had greater inhibitory activity compared to the extracts and rhizosphere soil obtained from its native ranges [55,65]. The total phenolics, flavones and saponins in *S. canadensis* in the invasive ranges were also greater than those in the native ranges [55,65,74]. These observations suggest that *S. canadensis* in the invasive ranges may enhance the competitive ability through the increased production of some of these compounds. The novel weapons hypothesis suggests that the competitive ability of the invasive plants is superior to that of the native plant species due to its allelochemicals. Those allelochemicals are new to the indigenous plant species, and those indigenous plants are susceptible to the compounds [34,91]. However, there has been no available information on the specific compounds of which the production is increased in *S. canadensis* in the invasive ranges, as well as the reasons for which those productions are increased in the invasive ranges.

*S. canadensis* showed the alteration of the composition of the arbuscular mycorrhizal population and suppressed the mycorrhizal colonization of the native plant species in the field and greenhouse experiments. The extracts of *S. canadensis* also suppressed the mycorrhizal colonization of the native plant species. These investigations suggest that certain compounds in the extracts may be involved in the suppression. The authors also indicated the involvement of allelochemicals in the suppression. Arbuscular mycorrhizal fungi are important mycorrhiza for the most territorial plants, and their colonization enhances the ability of the host plants to absorb mineral nutrients and water, as well as increase the defense functions against pathogen attacks and stress conditions [92,93,94]. The suppression of the colonization reduces the potential of the native plants for nutrient and water absorption, as well as the defense function, which may cause the reduction in growth and vigor of the native plant species.

All the literature described above indicates that *S. canadensis* may suppress the regeneration process of the native plant species directly by the suppression of their germination and growth, and indirectly by the degradation of the mycorrhizal fungal mutualism of the native plant species through its allelopathy. Therefore, the allelopathy of *S. canadensis* may contribute to its increasing competitive ability and make the plant invasive. However, it is necessary to identify the allelochemicals involved in the suppression of the fungal mutualism of the native plant species. Investigations into the specific inhibitory activity of the identified allelochemicals on the native plant species, as well as their concentrations in the rhizosphere soil and/or surrounding environments are necessary to evaluate the contribution of these allelochemicals to the invasiveness of *S. canadensis*.

## 3. Allelopathy of *S. altissima*

There has been limited information on the allelopathic activity of the root exudates, extracts and residues of *S. altissima,* unlike *S. canadensis*, as well as its allelopathy in the field conditions. The available information is the inhibitory effect of the *n*-hexane extracts of the underground parts of *S. altissima* on the growth of *Lactuca sativa* L. [95]. However, phytochemical investigations suggest that *S. altissima* contains polyacetylenes [96,97], monoterpene and sesquiterpenes [98], diterpenes [95,98,99,100,101,102], triterpenes [103,104], flavonoid glycosides [105,106] and polyphenols [107]. Some of them may act as allelopathic agents of the species.

### 3.1. Terpene

13*E*-7α-acetoxyl kolavenic acid (solidagonic acid) was isolated from the aqueous acetone extract of *S. altissima* [95,108], and the compound showed the growth inhibitory activity on the seedlings of *Lactuca sativa* L. and *Lolium multiflorum* Lam. [95,108]. 13*E*-kolavenic acid was also isolated from its *n*-hexane extracts [95]. Although 13*E*-kolavenic acid did not show any growth inhibitory activity, it exhibited antifungal activity and insect antifeedant activity [109,110]. These activities may help the species to protect itself from fungal pathogens and insect damage, as well as to establish its stands. It was reported that young rhizomes contained many more terpenes than old rhizomes, and α-pinene, β-pinene, limonene and germacrene D were identified in the rhizomes by GC-MS analysis [98]. α-Pinene, β-pinene, sabinene, myrcene, limonene, bornyl acetate and germacrene D were also found in the essential oil of *S. altissima*. However, the species was identified as a synonym of *S. canadensis* [33].

**Table 2:** Allelochemicals identified in *S. canadensis* and *S. canadensis* and their sources.

Chemical Class	Compound	*Solidado canadensis*		Reference	*Solidado altissima*			Reference
		Extract	Essential oil		Extract	Soil	Essential oil	
Fatty acid	**1**: *n*-Hexadecanonic acid	✓		[56]				
Polyacetylene	**2**: *cis*-Dehydromatricaria ester				✓	✓		[45,96,98,104]
	**3**: *trans*-Dehydromatricaria ester					✓		[45]
	**4**: (2Z,8Z)-10-Tigloyloxy matricaria ester				✓			[95]
	**5**: (2Z,8Z)-10-Angeloyloxy matricaria ester				✓			[95,96]
	**6**: Dehydromatricaria lactone				✓			[95,104,111]
	**7**: (4Z,8Z)-10-Trigloyloxy matricaria lactone				✓			[95,111]
	**8**: (4Z,8Z)-10-Angeloyloxy matricaria lactone				✓			[95]
Monoterpene	**9**: α-Pinene		✓	[83,84,85,86]	✓		✓	[33,98]
	**10**: β-Pinene		✓	[84]	✓		✓	[33,98]
	**11**: *trans*-Verbenol		✓	[83]				
	**12**: Limonene		✓	[83,84,85,86]	✓		✓	[33,98]
	**13**: Sabinene						✓	[33]
	**14**: Myrcene						✓	[33]
	**15**: Thymol		✓	[84]				
	**16**: Bornyl acetate		✓	[83]			✓	[33]
Sesquiterpene	**16**: β-Elemene		✓	[84]				
	**17**: (+)epi-Bicyclosesquiphellandrene		✓	[84]				
	**18**: β-Cadinene		✓	[84]				
	**19**: γ-Cadinene		✓	[84]				
	**20**: *δ-Cadinene*		✓	[84]				
	**21**: α-muurolene		✓	[84]				
	**22**: γ-muurolene		✓	[84]				
	**23**: Germacrene D		✓	[84,86]	✓		✓	[33,98]
	**24**: α-cubebene		✓	[84]				
	**25**: β-Cubebene		✓	[83,84]				
Diterpene	**26**: 13*E*-kolavenic acid				✓			[95]
	**27**: 13*E*-7α-acetoxyl kolavenic acid				✓			[95,108]
Polyphenol	**28**: Chlorogenic acid	✓		[76]				
Flavonoid	**29**: Kaempferol-3-*O*-D-glucoside	✓		[75]				
	**30**: Quercitrin	✓		[76,77]				
	**31**: Rutin	✓		[76]				

### 3.2. Polyacetylene

Several polyacetylenes, such as *cis*-dehydromatricaria ester (*cis*-DME), dehydromatricaria lactone, (2*Z*,8*Z*)-10-tigloyloxy matricaria ester, (4*Z*,8Z)-10-trigloyloxy matricaria lactone, (2*Z*,8*Z*)-10-angeloyloxy matricaria ester (methyl10-[(*Z*)-2-methyl-2-butenoyloxy]-(2*Z*,8*Z*)-2,8-decadiene-4,6-diynoate) and (4*Z*,8*Z*)-10-angeloyloxy matricaria lactone were isolated from the stems, roots and/or rhizomes of *S. altissima* [45,95,96,98,104,111]. Polyacetylenes in Asteraceae plants were reported to be synthesized from crepenynic acid, which is formed oleic acid with linoleic acid, introducing the first acetylene bond [95,112].

Among the polyacetylenes found in *S. altissima*, *cis*-DME, dehydromatricaria lactone and the (2*Z*,8*Z*)-10-angeloyloxy matricaria ester were reported to inhibit the coleoptile growth of *Panicum crus-galli* L. var. *frumentaceum* Trin. up to 77.4–93.5%, 81.7% and 80.0% that of the control, respectively, at the concentration of 1 ppm [96,111]. The (2*Z*,8*Z*)-10-Tigloyloxy matricaria ester also inhibited the growth of *Lolium multiflorum* Lam. [95]. However, *cis*-DME among those polyacetylenes was the most studied.

The bioactive concentration of *cis*-DME caused the growth inhibition, which was of 1–20 ppm in laboratory conditions [45,95,96,98,108,111,113]. *cis*-DME suppressed the germination of *Asclepias syriaca* L., *Ambrosia artemisiifolia* L. and *Miscanthus sinensis* Anderson; the germination and growth of *Poa pratensis* L. and *Oryza sativa* L.; and the growth of *Lactuca sativa* L. and *Panicum crus-galli* L. var. *frumentaceum* Trin. Their germination was inhibited up to 20–95% that of the control by 32–60 ppm *cis*-DME, and their growth was inhibited up to 10–56% that of the control by 48–100 ppm *cis*-DME (Table 3). *Asclepias syriaca* and *Miscanthus sinensis*, respectively, are competitive species with *S. altissima* in the native range of the USA and in the invasive range of Japan. *Ambrosia artemisiifoli* and *Poa pratensis* are the invasive plant species from North America, Europa and North Asia [45,98,114,115]. However, the effectiveness of *cis*-DME on those plant species was not evidently different.

The concentration of *cis*-DME was reported to be of 250–400 ppm in the roots of *S. altissima,* of 6.3 ppm in the soil under *S. altissima* [45] and of 0–17.3 ppm in the soil [116]. The concentrations in the soil varied depending on the soil properties and microbial activities [117,118,119]. The existence of *cis*-DME in the soil under *S. altissima* suggests that *cis*-DME may be released from *S. altissima* into the soils through the root exudation, rainfall leachates and/or the decomposition processes of plant residues. In addition, considering the bioactive concentration (1–20 ppm) described above and the soil concentration (0–17.3 ppm) of *cis*-DME, which is the same in laboratory conditions, the *cis*-DME in the soil may have some ecological function.

*trans*-DME was also in the soil under *S. altissima*, and its inhibitory activity was comparable of that of *cis*-DME [45]. When the ethanol solution (1%) of *cis*-DME was kept at 29 °C under 2000 lux for 2 days, 50% of *cis*-DME was isomerized into *trans*-DME, since the *cis*-form of the unsaturated ester is easily isomerized by light, pH or other conditions into the more stable *trans*-form [120]. In addition, *trans*-DME was not found in the roots and rhizomes of *S. altissima* [45]. Therefore, *cis*-DME may be isomerized by light and other environmental factors into *trans*-DME after the exudation from the plants into the soil. When we consider the involvement of DME in the allelopathy of *S. altissima*, the concentration of both *cis*-DME and *trans*-DME in the rhizosphere soil should be counted.

It was also reported that the aqueous extracts of the below-ground parts of *S. altissima* killed a pine wilt nematode (*Bursaphelenchus lignicolous* Mamiya and Kiyohara), and an active compound involved in it was determined as *cis*-DME. *cis*-DME at 10–11 ppm increased 50% of the molarity of the pine wilt nematode and a root-knot nematode (*Meloidogyne incognita* Kofild and White) [121]. The methanol extracts of the *S. altissima* rhizomes suppressed the hatching of fruit flies (*Drosophila melanogaster* Meigen), and an active compound was also determined as *cis*-DME [97]. The defense capacity of the invasive plants with natural enemies, such as pathogens, parasites and herbivores, contributes their establishment and naturalization into introduced ranges [37,122,123]. Therefore, the nematicidal and insecticidal activities of *cis*-DME may contribute to the establishment and naturalization of *S. altissima* in the introduced ranges.

### 3.3. Contribution of Allelopathy of S. altissima to Its Invasiveness

Unlike *S. canadensis*, the information on the allelopathic activity of *S. altissima* extracts, root exudates and residues is limited. However, polyacetylenes, monoterpene and sesquiterpenes, diterpenes, triterpenes and flavonoid glycosides were identified in its extracts and essential oil as allelopathic agents. Most potential compounds among them involved in the allelopathy may be *cis*-DME. *cis*-DME inhibited the germination and growth of several plant species at the concentration of 1–20 ppm [45,95,98,108,111,113]. Its concentration in the roots of *S. altissima* and in its rhizosphere soil was of 250–400 ppm and 0–17.3 ppm, respectively [45,116]. These observations suggest that *cis*-DME may be released into the rhizosphere soil by root exudation, rainfall leachates and/or decomposition processes of plant residues. *trans*-DME was also formed by the isomerization of *cis*-DME in the soil, and its inhibitory activity was the same as *cis*-DME. Although there has been no information of the concentration of *trans*-DME in the rhizosphere soil of *S. altissima*, the concentration of only *cis*-DME in several soils was over the concentration which was able to cause the growth inhibition [45,116]. In addition, *cis*-DME possesses nematicidal and insecticidal activities [97,113,121,124] Some flavonoids were also identified in the aerial parts of *S. canadensis* [78]. 13*E*-kolavenic acid also showed antifungal activity and insect antifeedant activity [109,110]. Therefore, these compounds including *trans*-DME may be involved in allelopathy and/or defense function against natural enemies, such as fungal pathogens, parasites and herbivores, and may contribute to their establishment and naturalization into the introduced ranges. However, it is necessary to determine the concentration of *trans*-DME in the rhizosphere soil of *S. altissima*, as well as to investigate the allelopathy of *S. altissima* in greenhouse and field conditions. It is also worth investigating the effect of *S. altissima* on the arbuscular mycorrhizal colonization of the native plant species due to the fact that *S. altissima* and *S. canadensis* are very close species.

## 4. Conclusions

*S. canadensis* and *S. altissima* are harmful invasive species naturalized in many countries, and form thick monospecific stands. Based on the literature, both species are allelopathic. The root exudate, residues, extracts, essential oil and rhizosphere soil of *S. canadensis* showed allelopathic activity on several plant species, including native plants, and suppressed the arbuscular mycorrhizal mutualism of the native plant species. Several allelochemicals were also identified in its extracts and essential oil. The concentrations of total phenolics, total flavones and total saponins in the rhizosphere soil of *S. canadensis* obtained from the invasive ranges were greater than those obtained from the native ranges. Therefore, the allelopathy of *S. canadensis* may contribute the interruption of the regeneration process of native plant species directly through the suppression of their germination and growth, and indirectly through the suppression of the mycorrhizal fungal mutualism of the native plant species. Several allelochemicals of *S. altissima* were also identified in its extracts and essential oil. Among them, *cis*-DME inhibited the germination and growth of several plant species at a concentration of 1–20 ppm, and its extractable concentration in the rhizosphere soil of *S. altissima* was of 0–17.3 ppm. *cis*-DME also exhibited nematicidal and insecticidal activities. *trans*-DME was formed by the isomerization of *cis*-DME in the soil, and its inhibitory activity was similar to that of *cis*-DME. Therefore, *cis*-DME, including *trans*-DME, may also cause the suppression of the regeneration process of the native plant species through the inhibition of their germination and growth. The allelopathy of *S. canadensis* and *S. altissima* may provide the species with competitive advantage against native plant species, and contribute to their invasiveness and naturalization.

## Figures and Tables

**Table 3:** Growth inhibitory activity of *cis*-dehydromatricaria ester.

*cis-*DME Concentration (ppm)	Target Plant	Germination	Growth	Reference
		(% of Control)		
32	*Asclepias syriaca*	5		[98]
48	*Poa pratensis*	20	50	[113]
50	*Oryza sativa*	88	10–15	[45]
50	*Ambrosia artemisiifolia*	25		[45]
50	*Miscanthus sinensis*	22		[45]
100	*Lactuca sativa*		30–56	[108]
100	*Panicum crus-galli*		18	[111]
